# Survival of Patients with Alcohol-Related Liver Disease Cirrhosis—Usefulness of the New Liver Mortality Inpatients Prognostic Score

**DOI:** 10.3390/diagnostics14222508

**Published:** 2024-11-09

**Authors:** Vera Matovic Zaric, Ivana Pantic, Sofija Lugonja, Tijana Glisic, Snezana Konjikusic, Iva Lolic, Nevena Baljosevic, Sanja Zgradic, Jasna El Mezeni, Marko Vojnovic, Marija Brankovic, Tamara Milovanovic

**Affiliations:** 1 Emergency Center, Gastroenterology and Hepatology Department, University Clinical Center of Serbia, 11000 Belgrade, Serbia; loliciva@gmail.com (I.L.); jocicnevena@gmail.com (N.B.); 2 Clinic of Gastroenterology and Hepatology, University Clinical Center of Serbia, 11000 Belgrade, Serbia; ilic.ivana04@gmail.com (I.P.); tijana.glisic78@gmail.com (T.G.); sanjazgradic@gmail.com (S.Z.); jasnavujisic@gmail.com (J.E.M.); marko.vojna@gmail.com (M.V.); alempijevic.tamara@gmail.com (T.M.); 3 Department of Internal Medicine, Division of Gastroenterology, General Hospital “Djordje Joanovic”, 23000 Zrenjanin, Serbia; prolesofija@gmail.com; 4 Faculty of Medicine, University of Belgrade, 11000 Belgrade, Serbia; manive23@gmail.com; 5 FEFA Faculty, Metropolitan University, 11000 Belgrade, Serbia; zansak@gmail.com; 6 University Hospital Medical Center Bežanijska Kosa, 11000 Belgrade, Serbia

**Keywords:** liver cirrhosis, survival, score

## Abstract

**Background/Objectives:** Alcohol can directly damage the liver, causing steatosis, steatohepatitis, cirrhosis, and hepatocellular cancer. The aim of this study was to examine 28-day survival in hospitalized patients with alcohol-related liver disease (ALD) cirrhosis, as well as to develop and validate a new survival prediction model. **Methods:** A total of 145 patients with ALD cirrhosis were included; 107 were diagnosed with acute decompensation (AD) and 38 with acute-on-chronic liver failure (ACLF). The new liver mortality inpatients (LIV-IN) score was calculated using the following variables: hepatic encephalopathy (HE), hepatorenal syndrome (HRS), ascites, systemic inflammatory response syndrome (SIRS), community-acquired infection (CAI), and fibrinogen. The diagnostic accuracy of the LIV-IN score was tested, along with the model for end-stage liver disease (MELD), model for end-stage liver disease-sodium (MELD-Na), albumin-bilirubin (ALBI), neutrophil-to-lymphocyte ratio (NLR), chronic liver failure consortium-C acute decompensation (CLIF-C AD), and chronic liver failure consortium-acute-on-chronic liver failure (CLIF-C ACLF). **Results:** Lethal outcome occurred in 46 (31.7%) patients. The mortality rate was higher in the ACLF group (*n* = 22, 57.9%) compared to the AD group (*n* = 24, 22.4%) (*p* < 0.01). The highest predictive power for short-term mortality was observed for the LIV-IN score (AUC 73.4%, *p* < 0.01). In patients with AD, the diagnostic accuracy of the CLIF-C AD score was better than for the LIV-IN score (AUC 0.699; *p* = 0.004, AUC 0.686; *p* = 0.007, respectively). In patients with ACLF, only the LIV-IN score had statistically significant discriminative power in predicting 28-day survival. **Conclusions:** The liver mortality inpatients prognostic score is a new, reliable prognostic model in predicting 28-day mortality.

## 1. Introduction

Alcohol consumption is associated with the occurrence of a large number of diseases, such as alcohol-related liver disease (ALD), arterial hypertension, cardiomyopathy, acute and chronic pancreatitis, gastritis, esophageal cancer, etc. [1]. According to World Health Organization (WHO) data, excessive alcohol consumption was the main cause of death of 3 million people in 2016 [2]. Alcohol may lead to direct toxic liver damage and cause liver steatosis, steatohepatitis, cirrhosis, and hepatocellular carcinoma (HCC). In Europe, the mortality rate due to ALD cirrhosis is 9.2/100,000 inhabitants [3]. In 2019, out of the total number of deaths due to liver disease, one-fourth was related to ALD cirrhosis [4]. Patients with decompensated liver cirrhosis have a 9.7 higher risk of death compared to the general population [5]. Infections, especially spontaneous bacterial peritonitis (SBP) and sepsis, as well as hepatorenal syndrome (HRS), increase the risk for mortality by 10–20 times. Hepatic encephalopathy (HE), variceal bleeding, and ascites are also associated with decreased survival [3].

According to the study of Tonon, M., et al., individuals with grade I ascites experienced higher rates of death, comorbidities, and systemic inflammation than those without ascites [6]. Another study showed that 0.6% and 14.9% of patients without ascites developed HRS and infections, respectively, while this number was higher in patients with ascites grade I [7]. Another useful indicator of advanced liver disease is systemic inflammatory response syndrome (SIRS), which is linked to adverse outcomes. It can result in consequences like HE, variceal bleeding, HRS, and further liver damage [8].

Active alcohol use, however, has been shown to be the primary predictor of survival in individuals with ALD cirrhosis because it may enhance liver inflammation and bacterial translocation, which in turn may increase intrahepatic resistance and multiorgan failure [9].

Patients diagnosed with decompensated liver cirrhosis are more prone to bacterial infection due to immune dysfunction, increased gut permeability, intestinal bacterial overgrowth, gut dysbiosis, etc. According to previously published studies, approximately 32–40% of inpatients had community-acquired (CA) or healthcare-associated infections (HAIs), which further decreased short-term survival [10].

In daily clinical practice, the most frequently used prognostic scores are the model for end-stage liver disease (MELD), the model for end-stage liver disease-Sodium (MELD-Na), and the Child–Pugh score [11,12]. Since 2013, the chronic liver failure-sequential organ failure assessment (CLIF-C SOFA) score for critically ill patients has been in use, along with the chronic liver failure consortium-acute-on-chronic liver failure (CLIF-C ACLF) score for patients with ACLF and the chronic liver failure consortium-C acute decompensation (CLIF-C AD) for patients with acute decompensation without ACLF [13]. However, these two scores did not show the expected superiority in relation to the MELD, MELD-Na, and Child–Pugh scores in all studies [14]. Jalan et al. have shown that patients with a CLIF-C AD score ≥60 had similar 90-day mortality as patients with ACLF grade I [15]. The accuracy of the CLIC-C ACLF score was also not ideal regarding the survival predictions in ICU patients, probably due to the ceiling effect of serum bilirubin, INR, and creatinine [16,17,18].

One well-known feature of liver cirrhosis is chronic systemic inflammation, mainly related to bacterial translocation [19]. The neutrophil-to-lymphocyte ratio (NLR) is a simple, well-known marker of systemic inflammation, with previously documented usefulness as a prediction tool in patients with liver cirrhosis for both acute decompensation (AD) and acute-on-chronic liver failure (ACLF) [20].

Another novel prognostic score is albumin-bilirubin (ALBI), which can reflect early liver function deterioration. Firstly, it was used for patients with HCC, and later it was proven as a good survival predictor for other non-malignant chronic liver diseases [21].

Fibrinogen, produced by the liver, is an important coagulation factor for primary and secondary hemostasis, and a decreased level is associated with poor outcome in patients with ACLF [22].

Ascites, HE, creatinine, sodium, bilirubin, albumin, and age are well-known survival predictors that were included in the established predictive models, as mentioned earlier, but in our study we wanted to highlight the significance of often overlooked variables like SIRS, fibrinogen, and infections. Also, for decompensated liver cirrhosis, the crucial step is timely differentiation between HRS and other types of acute kidney injury because HRS is associated with a worse prognosis [23].

Based on this consideration, the present study aimed to investigate 28-day mortality in patients with ALD cirrhosis, as well as identify factors associated with decreased short-term survival. Moreover, this study investigated the diagnostic accuracy of the newly created original liver mortality inpatients (LIV-IN) score and the following “old“ prognostic scores MELD, MELD-Na, ALBI, NLR, and CLIF-C AD in patients with acute decompensation, and CLIF C-ACLF in patients diagnosed with ACLF.

## 2. Materials and Methods

### 2.1. Study Design and Inclusion Criteria

A prospective cohort observational study was conducted at the Gastroenterology and Hepatology Intensive Care Unit of the Emergency Center and the Semi-intensive Care Unit of the Clinic for Gastroenterohepatology, University Clinical Center of Serbia, Belgrade The study included hospitalized subjects with ALD cirrhosis, who were treated in our institution from November 2022 to August 2023.

The diagnosis of ALD cirrhosis was made based on anamnestic and/or hetero-anamnestic data on long-term harmful alcohol consumption, as well as based on typical laboratory analyses, clinical signs, radiological features, and after excluding other viral, immunological, and metabolic causes of liver disease. Patients who underwent transjugular intrahepatic portosystemic shunts (TIPS) or liver transplantation were excluded. Other exclusion criteria were as follows: age < 18 years, HCC, acute or chronic decompensation of another extrahepatic organ that may be related to multiorgan failure, extrahepatic cancers, severe trauma, human immunodeficiency virus (HIV) infection, and pregnancy. All patients were followed for 28 days. The study was approved by the Ethics Committee of the University Clinical Center of Serbia and was conducted according to the principles of the Helsinki Declaration (protocol code: 17/4; date of approval: 26 January 2023).

### 2.2. Data Collection

The following data were obtained from the patient’s medical records: age, gender, presence of ascites, HRS, HE, upper gastrointestinal bleeding (UGIB), and SIRS, as well as body temperature, respiratory rate, oxygen saturation (SpO2), arterial pressure, and data regarding active alcohol consumption. In each patient, the initial diagnostic work-up included a chest X-ray, abdominal ultrasound, complete blood count, comprehensive metabolic panel, and microbial cultures (venous blood and urine). In cases in which diagnostic paracentesis could be performed, peritoneal fluid was sampled and sent for biochemical, cytological, and microbial analyses.

According to national guidelines for the diagnosis and treatment of alcohol use disorder (AUD), issued by the Serbian Ministry of Health, active and excessive alcohol consumption was defined as alcohol intake >14 standard drinks weekly, or >4 standard drinks on one occasion in men, and >7 standard drinks weekly, or >2 standard drinks on one occasion in women, at least during the last three months. One standard drink is defined as containing 13 g of alcohol (one small bottle of beer (330 mL, 5% of ethanol), one glass of wine (120 mL, 12% of ethanol), and one glass of spirits (40 mL, 40% of ethanol) [24].

According to the European Association for the Study of the Liver (EASL) criteria, ascites was graded as follows: “mild” or grade I ascites—if it was detectable only by ultrasound; “moderate” or grade II—if the clinical examination showed symmetrical abdominal distension with positive clinical signs for ascites; and “large” or grade III—a large amount of free fluid with visible abdominal distension [25]. EASL criteria were also applied for the diagnosis of HRS and SBP [25].

The West Heaven criteria were used for grading HE. Patients with minimal HE and grade I HE according to the West Haven criteria were classified as covert HE, while patients with HE grades II, III, and IV according to the West Haven criteria were classified as overt HE. In patients who did not meet the West Heaven criteria for grade II, the animal naming test was used to establish the diagnosis of covert encephalopathy. In the animal naming test, the patients were asked to name as many animals as possible within 1 min. If the subjects named less than 10 animals in the specified period, the diagnosis of covert hepatic encephalopathy was made [26].

Patients diagnosed with infection on admission or within the first 48 h of admission were considered to have a CAI. If the infection was proven by diagnostic processing after 48 h, the patient was considered to have a HAI.

The following criteria were used to diagnose a urinary tract infection (UTI): abnormal urine sediment (>10 WBC per microscopic field of view) with a positive urine culture (>100.000 colony forming unit (CFU)). Based on the clinical findings and a chest X-ray that observed inflammatory infiltrates in the lung, pneumonia was diagnosed. The term spontaneous bacteremia was defined as the presence of a positive blood culture without an evident primary focus of infection.

All patients who were hospitalized due to UGIB underwent an upper endoscopy. Based on the upper endoscopy findings, bleeding was categorized as variceal, ulcer, or other, in cases when other pathological changes were identified in the upper portion of the GI area, such as portal hypertensive gastropathy, Mallory–Weis syndrome, gastric antral venectasia, Dilafou lesion, etc.

The patients were diagnosed with SIRS if they met two of the following four criteria: 1. body temperature > 38 °C or <36 °C, 2. heart rate >90/min, 3. respiratory rate >20/min, and 4. WBC > 12 × 10^9^ or <4 × 10^9^.

The term AD of liver cirrhosis was considered in cases of complications of the underlying disease, such as hepatic encephalopathy, ascites, variceal bleeding, bacterial infection, or as a combination of the aforementioned complications.

The term ACLF was used in cases of severe acute decompensation of liver cirrhosis with functional failure of one or more of the following six organ systems: liver, kidney, brain, coagulation, circulation, and respiratory system. To stratify the subjects, the European Association for the Study of the Liver (EASL)-chronic liver failure (CLIF) consortium (CLIF-C) ACLF criteria were used [27].

The following criteria were used for the diagnosis of organ failures: HE grade III or IV for brain failure; SpO2/FiO2 ≤ 214 for lung failure; need for vasopressors for circulatory failure; total bilirubin ≥ 12 mg/dL for liver failure; INR ≥ 2.5 for coagulation failure; and creatinine ≥ 2.5 mg/dL or need for renal replacement therapy for kidney failure [27].

According to the previously recorded data, the following prognostic scores were calculated using MD+Calc (available online: https://www.mdcalc.com (accessed on 15 Septembar 2024)): MELD, MELD-Na, NLR, ALBI. In patients with acute decompensation without ACLF, the CLIF-C AD score was calculated, as well as the CLIF-C ACLF score in patients with ACLF diagnosis. Online CLIF-C OF/ AD/ ACLF score calculator was used for the last two above-mentioned scores (https://efclif.com/research-infrastructure/score-calculators/clif-c-of-aclf-ad/- accessed on 15 September 2024).

In the ACLF group, if one organ failure system was identified, the patients were further classified into grade I, for failure of two organ systems into grade II, and three or more organ failures into grade III.

### 2.3. Follow-Up

All patients were followed up for 28 days. Subjects lost in the follow-up for different reasons were excluded from the present study. Also, if a hepatic or extrahepatic tumor diagnosis was made during the mentioned follow-up period, the patients were excluded from the study. In the case of a fatal outcome, the cause of death was recorded.

### 2.4. Statistical Analysis

Numerical variables with normal distribution were expressed as mean ± standard deviation (SD), as well as median with a 25–75% interquartile range in cases where the criteria for normal distribution were not fulfilled. Nominal and ordinal variables are presented as absolute numbers and percentages. The Kolmogorov–Smirnof test was used to check the distribution normality. For the analysis of categorical variables, r × k contingency tables, Fischer’s test, and Person Chi-square test were used. Numerical data with normal distribution were analyzed using the Student’s *t*-test and ANOVA, and if the distribution was not normal, the Mann–Whitney test was used. The ROC curve was used to analyze the clinical accuracy of the prognostic scores. The identification of risk factors for the occurrence of a lethal outcome was carried out using logistic regression. Statistical significance is defined as *p* < 0.05. Statistical analysis was performed using the statistical software IBM SPSS, version 20 (Chicago, IL, USA).

## 3. Results

### 3.1. Demographic and Clinical Characteristics of Patients with ALD Cirrhosis

**Figure 1:**
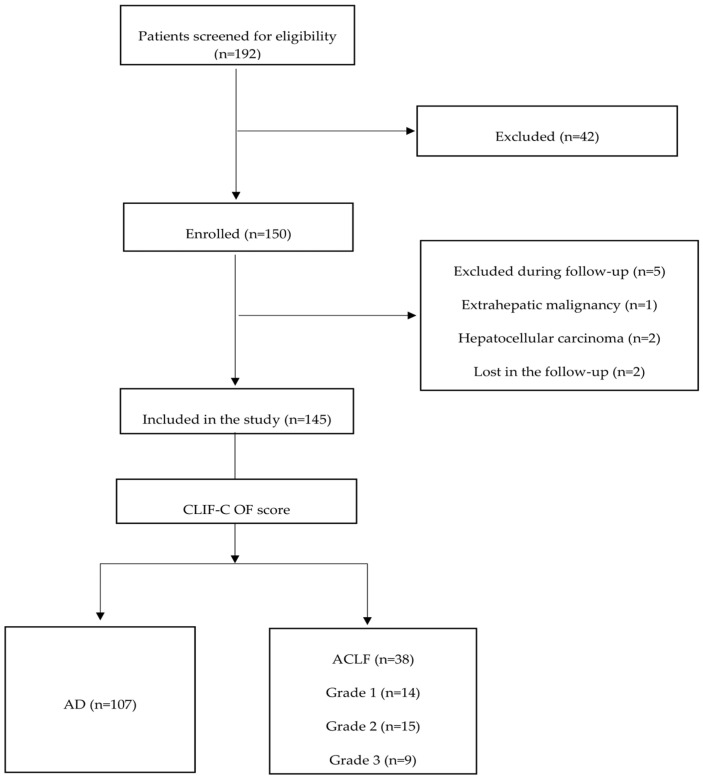
Study inclusion flow-chart.

After the initial diagnostic work-up, a total of 150 patients who met the inclusion criteria were enrolled in the study. During the following period, 5 patients were excluded due to different reasons, and the final number of all included patients in our study was 145. Based on the CLIF-C-OF score, the patients were further divided into two groups: acute decompensation (AD) (*n* = 107) and ACLF (*n* = 38) (Figure 1).

Baseline demographic and clinical characteristics are presented in Table 1. Among the patients included in this study, 129 (89%) were male, with a mean age of 56.42 ± 10.87 years. No difference in age was recorded between patients with AD and ACLF, while the frequency of the female gender was significantly higher in ACLF patients compared to the AD group (*p* = 0.03). On admission, 123 (84.8%) patients were actively drinking alcohol. Eighty-three (57.2%) patients were admitted to the ICU. ICU admission was more common in ACLF compared to AD patients (73.7% vs. 51.4%, *p* = 0.02).

Ascites was present in 80.7% of the patients. Compared to patients with AD, patients with ACLF experienced ascites more frequently (*p* = 0.009). Almost all patients in the ACLF group had ascites, and the most frequent was ascites grade III (*n* = 25; 69.4%).

Ninety-seven patients (66.9%) were diagnosed with HE, among them 87.6% (*n* = 85) of patients had overt, and 12 (12.4%) had covert hepatic encephalopathy. Hepatic encephalopathy was more commonly detected in ACLF patients compared to those with AD (86.8% vs. 59.8%, *p* = 0.02). The overt form of hepatic encephalopathy was more frequently reported in AD patients (90.6% vs. 81.8%, *p* = 0.04).

Upper gastrointestinal bleeding was seen in 55 (37.9%) cases. Thirty-one patients (56.4%) were diagnosed with variceal bleeding, 4 (7.3%) with ulcer bleeding, and other causes of UGIB were seen in 20 (36.4%) patients. There were no differences in terms of UGIB between the compared groups.

HRS were diagnosed in 19 patients; among them, 9 (8.5%) were in AD and 10 (26.3%) were in the ACLF group (*p* = 0.01). SIRS was seen in 38 (26.2%) patients, and it was more frequent in the ACLF group (39.47% vs. 21.49%, *p* = 0.03).

The following organ failures were observed in patients with ACLF: circulatory (*n* = 11; 28.9%), respiratory (*n* = 12; 31.6%), liver (*n* = 16; 42.1%), kidney (*n* = 10; 26.3%), coagulation (*n* = 9; 23.7%), and brain (*n* = 20; 52.6%). Fourteen patients (36.8%) were diagnosed with ACLF grade I, fifteen (39.4%) with ACLF grade II, and nine (23.6%) with ACLF grade III.

Baseline laboratory analyses are presented in detail in Table 2. In the ACLF group, the following laboratory analyses were significantly higher than in the AD group: WBC, total bilirubin, creatinine, CRP, fibrinogen, procalcitonin, PT, and INR (*p* < 0.01). Serum albumin level was statistically lower in the ACLF group (*p* < 0.05).

Forty-five patients (31.03%) were diagnosed with CAIs. There were no statistical differences in the frequency of CAIs among AD and ACLF groups (*n* = 32 (29.9%); *n* = 13 (34,2%), respectively, *p* = 0.07). The most common CAI was UTI (*n* = 21; 46.7%), followed by pneumonia (*n* = 14; 31,1%). Four patients (8.9%) were diagnosed with pneumonia and UTI, and bacteremia was seen in another four cases (8.9%). The most frequent CAI in the AD group was UTI (*n* = 21; 46,7%), while pneumonia was the most common in the ACLF group (*n* = 6; 46.2%) (Table 3).

Health-care-associated infections were found in 63 patients (43.4%), among them 41 (38.3%) were from the AD group and 22 (57.9%) were from the ACLF group (*p* = 0.05). As shown in Table 3, the following types of infection were diagnosed: UTI (*n* = 35; 55.6%), bacteremia (*n* = 20; 31.7%), pneumonia (*n* = 5; 7.9%), SBP (*n* = 2; 3.2%), and combined UTI and pneumonia (*n* = 1; 1.6%). Urinary tract infection was the most frequent primary infection focus in the AD group (*n* = 25; 61%). Bacteremia, as well as UTI, were the most frequently observed HAI in the ACLF group.

### 3.2. Twenty-Eight Days Survival of Patients with ALD Cirrhosis

During the 28-day follow-up, a fatal outcome occurred in 46 (31.7%) patients. The mortality rate was significantly higher in the ACLF group (*n* = 22, 57.9%) compared to the AD group (*n* = 24, 22.4%) (*p* < 0.01). In the ACLF group, the mortality rate in terms of ACLF grade was as follows: grade I, *n* = 6 (42.9%); grade II, *n* = 8 (57.1%); grade III, *n* = 8 (88.9%).

The main causes of death are presented in Table 4. Liver-related death occurred in 15 (32.5%) patients, while the rest was due to another extrahepatic reason. The most frequently observed cause of death was heart failure (*n* = 17; 37%), followed by lung failure (*n* = 13; 28.3%). Liver-related deaths due to variceal bleeding, infections, and other liver-related complications in the AD group were seen in nine (37.5%) patients, while this number was lower in the ACLF group (*n* = 6, 27.2%).

The main predictive factors associated with 28-day mortality in univariate analysis were as follows: HE (OR 4.912, *p* = 0.01), CAIs (OR 2.3, *p* = 0.023), ascites (OR 7.836, *p* = 0.007), HRS (OR 6.040, *p* = 0.001), SIRS (OR 8.775, *p* < 0.01), and ACLF (OR 7.25, *p* < 0.01). When we further enrolled all these statistically significant variables from univariate analyses into multivariate analyses, only the following four remained statistically significant: HE (OR 3.63, *p* = 0.04), ascites (OR 6.896, *p* = 0.03), SIRS (OR 9.323, *p* < 0.01), and ACLF (OR 3.539, *p* = 0.013) (Table 5).

In univariate analysis, gastrointestinal and variceal hemorrhage had no association with 28-day survival (*p* = 0.369 and 0.95, respectively).

We also investigated organ failures as predictors of 28-day mortality. The following organ failures were significantly associated with 28-day mortality: respiratory failure (*p* = 0.04, OR 4.12), coagulation failure (*p* = 0.03, OR 4.59), and brain failure (*p* < 0.01, OR 15.74). In multivariate analysis, none of these three organ failures are statistically significant predictors of 28-day mortality.

### 3.3. Prediction of 28-Day Mortality Based on Prognostic Scores-Formulation of the New LIV-IN Prognostic Score

According to the previously mentioned predictive factors, a new original prognostic score named liver mortality inpatients (LIV-IN) for inpatients with decompensated ALD cirrhosis was created. In this predictive model, we enrolled the following variables: CAIs, HE, ascites, HRS, SIRS, and fibrinogen. Variables were scored as follows:

1. Community-acquired infection: Yes (1 point); No (0 point);
2. Hepatic encephalopathy: Yes (1 point); No (0 point);
3. Ascites; without (0 point); mild (1 point); moderate (2 points); large (3 points);
4. HRS: Yes (1 point); No (0 point);
5. SIRS: Yes (1 point); No (0-point);
6. fibrinogen (g/L).

The score was calculated as a sum of all points with serum fibrinogen value.

The present study examined the clinical accuracy of the LIV-IN predictive model and previously known following scores in predicting 28-day mortality: MELD, MELD-Na, ALBI, NLR. For patients with AD without ACLF, we also evaluated the CLIF-C AD score, as well as the CLIF-C ACLF score for patients with ACLF.

Patients with ACLF had statistically higher MELD and MELD-Na scores, as well as LIV-IN score (*p* < 0.01). There were no differences in NLR scores among groups. The ALBI score was significantly lower in the ACLF group (Table 6).

**Figure 2:**
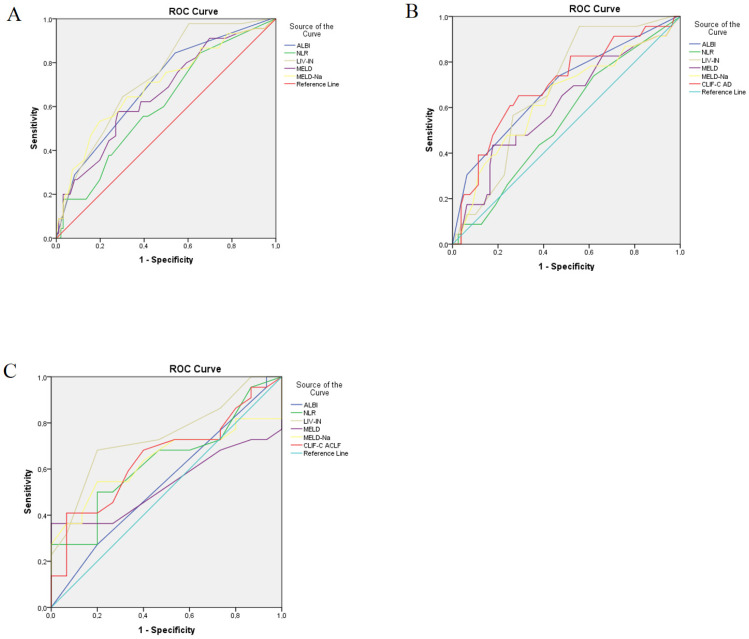
Receiver operating characteristic (ROC) curve for discriminative ability of prognostic scores in predicting 28-day mortality. (**A**) ROC curve for discriminative ability of prognostic scores in predicting 28-day mortality in patients with ALD cirrhosis. (**B**) ROC curve for discriminative ability of prognostic scores in predicting 28-day mortality in patients with AD. (**C**) ROC curve for discriminative ability of prognostic scores in predicting 28-day mortality in patients diagnosed with ACLF. Abbreviations: MELD—model for end-stage liver disease; MELD-Na—model for end-stage liver disease-sodium; ALBI—albumin-to-bilirubin ratio; NLR—neutrophil-to-lymphocyte ratio; LIV-IN—liver mortality inpatients; CLIF-C ACLF—chronic liver failure consortium-acute-on-chronic liver failure; CLIF-C AD—chronic liver failure consortium-C acute decompensation.

The statistically significant discriminative power of five evaluated scores (LIV-IN, MELD, MELD-Na, NLR, and ALBI) was observed in predicting 28-day mortality in patients with ALD cirrhosis. The ROC curve is shown in Figure 2A and the summary results in Table 7. According to the results of the ROC curve, the highest predictive power was observed for the LIV-IN score (AUC 73.4%). The estimated ROC curve for MELD, MELD-Na, ALBI, and NLR scores suggests that their predictive power is 66.1%, 69.6%, 69.6%, and 61%, respectively.

We further evaluated the clinical accuracy of the prognostic scores in predicting 28-day mortality in the AD group. The following scores in this study group were examined: LIV-IN, MELD, MELD-Na, ALBI, NLR, and CLIF-C AD. Statistically significant discriminative power was as follows: LIV-IN score (*p* = 0.007, AUC 0.686, CI 0.577–0.795), CLIF-C AD (*p* = 0.004, AUC 0.699, 95% CI (0.574–0.824)), ALBI (*p* = 0.007, AUC 0.685, 95% CI (0.556–0.814)), and MELD-Na (0.04, AUC 0.635, 95% CI (0.500–0.770)). An ROC curve of the examined scores is shown in Figure 2B.

In the ACLF group, we analyzed LIV-IN, MELD, MELD-Na, NLR, ALBI, and CLIF-C ACLF scores (Figure 2C). Only the LIV-IN score showed statistically significant discriminative power in predicting 28-day mortality in patients with ACLF (*p* = 0.01, AUC 0.742, 95% CI (0.583–0.902), Sn 72.7% and Sp 53.3% for cut-off value of 6.5).

## 4. Discussion

The primary aim of the present study was to analyze the 28-day survival of hospitalized patients with decompensated ALD cirrhosis. During the 28-day follow-up, a lethal outcome occurred in 46 (31.7%) patients. Lethal outcome was significantly more common in the ACLF group (*n* = 22, 57.9%) compared to the AD group (*n* = 24, 22.4%). In the ACLF group, increases in ACLF grades were followed by increases in mortality, and these results are in accordance with previously published data [28,29,30]. All patients who were diagnosed with ACLF in our study had cirrhosis, which may also affect survival, as shown in the study of Thanapirom, K., et al. [31]. Thanapirom, K., et al. also suggested that patients with ACLF with cirrhosis had better outcomes compared to ACLF patients without cirrhosis, explained by an inappropriate immune response that could result in less organ damage [31]. The 28-day mortality in acute decompensation of liver cirrhosis regardless of cirrhosis etiology was 20.8% in previously published data, which was similar to the results of the present study [32]. However, the results are not unique, so in the study that was conducted on patients who were hospitalized through emergency admission it was observed that in-hospital mortality was lower and amounted to 15.9%. In addition, the average value of the MELD score in that study was also lower compared to our results [33].

Alcohol consumption, liver cirrhosis itself, as well as the use of drugs for the treatment of decompensated liver cirrhosis, can lead to heart failure and malignant rhythm disorders, and the same can lead to death [34,35]. Analyzing the causes of death in our population, it was observed that heart failure with malignant rhythm disorder is one of the most common causes of death. The present study identified other causes of death as respiratory failure due to pulmonary embolism, acute respiratory distress syndrome, pneumonia, then sepsis, bleeding with shock, etc. The leading causes of death in patients with ACLF were hemorrhagic shock and respiratory failure, as shown in recently published data by Liu, L.X., et al. [36]. The development of acute pancreatitis with necrosis, heart failure, heart attack, mesenteric thrombosis, and renal failure can directly lead to death in subjects with ALD cirrhosis [37]. The above data imply that a multidisciplinary approach is needed in the treatment of critically ill patients with ALD cirrhosis.

In daily clinical practice, it is very important to identify risk factors for fatal outcomes. The main predictive factors associated with 28-day mortality in univariate analysis in our study were as follows: HE, community-acquired infections, HRS, ascites, SIRS, and ACLF. When we further enrolled all these statistically significant variables from univariate analysis into multivariate analyses, only the following four remained statistically significant: HE, ascites, SIRS, and ACLF. In the study by Trifan et al., which was conducted on more than 1000 subjects with ALD cirrhosis, it was observed that variceal bleeding, infection, SBP, sepsis, HE, ascites, HCC, and HRS were associated with an unfavorable outcome of the first hospitalization [3]. Other studies identified MELD score, lactates, infection, albumin, and CRP and CRP/albumin ratio as independent predictors of in-hospital mortality [33,38]. Also, a study examining only patients with ACLF showed that independent predictors of 28-day mortality in patients with ACLF were MELD score > 26, ACLF grade III, need for ventilation, shock, and use of hemodialysis [29,30]. Based on all previous studies, as well as the results of our research, it can be concluded that hospitalized patients with ALD cirrhosis are a real challenge in treatment and follow-up.

Nowadays, mathematical prognostic scores are used in all areas of medicine in daily clinical practice for easier assessment of patients’ prognoses. In hepatology, CP and MELD are the two scores that were first introduced into clinical practice, and they are still very valuable tools, even with slight modifications (e.g., MELD-Na). In recent years, based on the results of the CANONIC study, two more scores have been used in clinical practice. The CLIF-C ACLF score is a clinically relevant, validated score that is superior in predicting mortality compared to the MELD and MELD-Na score for patients with ACLF, while the CLIF-C AD score is more relevant than other scores for predicting mortality in hospitalized patients with acute decompensation [15]. The calculation of the ALBI score uses bilirubin and albumin and therefore shows the degree of liver damage. It has been shown that it can be a reliable score for predicting survival in HCC but also for patients with ACLF [36,39].

In this study, we evaluated the discriminative power of MELD, MELD-Na, ALBI, and NLR scores in predicting 28-day mortality. Also, we created a new original predictive model named LIV-IN score for ALD cirrhosis based on the following variables: HE, ascites, SIRS, HRS, community-acquired infection, and fibrinogen. According to the results of the ROC curve, the highest predictive accuracy was observed for the new LIV-IN prognostic score (AUC 73.4%). The estimated ROC curve for MELD, MELD-Na, ALBI, and NLR scores suggests that their predictive power is 66.1%, 69.6%, 69.6%, and 61%.

Diagnostic accuracy of MELD and MELD-Na scores were lower in our study compared to the results of previously published data [40,41,42].

In the group of patients with acute decompensation, we examined all the aforementioned scores along with the CLIF-C AD score. Statistically significant discriminative power revealed the following: LIV-IN score (AUC 0.686), CLIF-C AD (AUC 0.699), ALBI (AUC 0.685), and MELD-Na (AUC 0.635).

In the ACLF group, only the LIV-IN score showed statistically significant discriminative power in predicting 28-day mortality in patients with ACLF (*p* = 0.01, AUC 0.742), which may be due to a small number of included patients with ACLF.

Patients with AD, particularly those with ACLF, have a conspicuous systemic inflammatory response and a high chance of dying [43]. However, even though SIRS was described in 42.7% of patients with decompensation of liver cirrhosis in the previously published data and was associated with alcohol-related liver disease, its diagnostic value in this specific group of patients remains questionable due to the influence of beta blockers, hypersplenism, and hyperventilation in liver cirrhosis [44]. In the present study, 38 (26.2%) patients were diagnosed with SIRS, and the same was more frequent in the ACLF group. The current study identified SIRS as a predictive factor of short-term mortality, and because of that, it was one of the variables that was included in the calculation of the LIV-IN score.

As mentioned earlier, one of the variables from the LIV-IN score was community-acquired infection, and this is the first score that implemented bacterial infection in the predictive models. In addition to causing liver disease, alcohol consumption also increases the risk of systemic bacterial infections. It acts on T-cells in the skin and leads to infections caused by Staphylococcus aureus [45,46]. In addition, alcohol directly leads to mucosal damage at the level of the gastrointestinal tract and bacterial overgrowth, which leads to increased bacterial translocation [46]. Alcohol consumption also affects the alveolar epithelium and increases the risk of pneumonia [46]. On the other hand, liver cirrhosis is characterized by immune dysfunction and excessive activation of pro-inflammatory cytokines, malnutrition, which makes these patients susceptible to infections [47,48,49]. Although the cause of acute decompensation, especially ACLF, is very often a bacterial infection, the diagnosis is not always easy due to the present impaired liver function, pronounced pro-inflammatory response, hypersplenism, abdominal distension, negative cultures, and altered state of consciousness, and early recognition and treatment of the infection is crucial [49].

In our study, 45 patients (31.03%) were diagnosed with community-acquired infections, and 63 patients (43.4%) were diagnosed with healthcare-associated infections. The primary focuses of infection were as follows: UTI, pneumonia, spontaneous bacteremia, and SBP.

## 5. Conclusions

Hospitalized patients with ALD cirrhosis have a high 28-day mortality rate. According to the results of our study, the main cause of death was cardiovascular event, respiratory failure, and liver-related deaths. In univariate analysis, the predictive factors of lethal outcome were as follows: community-acquired infections, ascites, HE, HRS, SIRS, and ACLF. In multivariate analysis, only HE, ascites, SIRS, and ACLF were statistically significant. The new LIV-IN score for the prediction of 28-day mortality was created and validated. The diagnostic accuracy of this new score was higher than the diagnostic accuracy of the MELD, MELD-Na, ALBI, NLR, and CLIF-C ACLF scores. This is the first score that implemented fibrinogen, CAIs, and SIRS in a survival predictive model. We hope that the LIV-IN score accuracy will be validated in future studies on a larger number of patients, and that it will be useful for clinicians in everyday clinical practice.

## Figures and Tables

**Table 1:** Demographic and clinical characteristics of patients with alcoholic liver cirrhosis.

	Total	AD	ACLF	*p*
*n*	145	107	38	
Age (years) 🗶	56.42 ± 10.87	57.07 ± 10.81	54.6 ± 10.98	0.232
Gender (m/f) (%)	129/16 (88.9/11.3)	99/8 (92.5/7.5)	30/8 (78.9/21.1)	0.03
Active alcohol consumption (Yes/No) (%)	123/22 (84.8/15.2)	88/19 (82.8/17.8)	35/3 (92.1/7.9)	0.192
ICU Yes/No (%)	83/62 (57.2/42.8)	55/52 (51.4/48.6)	28/10 (73.7/26.3)	0.02
Ascites Yes/No (%)	117/28 (80.7/19.3)	81/26 (75.7/24.3)	36/2 (94.7/5.3)	0.009
Grade *n* (%)				
I	32 (27.3)	27 (33.3)	5 (13.9)	
II	30 (25.64)	24 (29.6)	6 (16.7)	
III	55 (47)	30 (37)	25 (69.4)	
HE Yes/No (%)	97/48 (66.9/33.1)	64/43 (59.8/40.2)	33/5 (86.8/13.2)	0.02
Type:				
Covert (*n*/%)	12 (12.4)	6 (9.4)	6 (18.1)	
Overt (*n*/%)	85 (87.6)	58 (90.6)	27 (81.8)	
HRS Yes/No (%)	19/125 (13.2/86.8)	9/97 (8.5/91.5)	10/28 (26.3/3.7)	0.01
SIRS Yes/No (%)	38/107 (26.2/73.8)	23/84 (21.49/82.24)	15/23 (39.47/60.5)	0.03
UGIB Yes/No (%)	55/90 (37.9/62.1)	43/64 (40.2/59.8)	12/26 (31.6/68.4)	0.43
Bleeding focus *n* (%)				
Variceal	31 (56.4)	26 (60.45)	5 (41.67)	
Ulcer	4 (7.3)	3 (6.97)	1(8.3)	
Other	20 (36.4)	14 (32.5)	6 (50)	

AD—acute decompensation; ACLF—acute-on-chronic liver failure; ICU-intensive care unit; HE—hepatic encephalopathy; SIRS—systemic inflammatory response syndrome; UGIB—upper gastrointestinal bleeding; HRS—hepatorenal syndrome; *n*—number of patients; m—male; f—female; 🗶—mean ± SD.

**Table 2:** Baseline laboratory analyses.

Variables	Total Study Group	AD	ACLF	*p*
WBC (×10^9^/L) ^a^	9.6 (7.1–15.2)	8.2 (6.8–12.35)	14.6 (10.6–17.63)	<0.01
Plt (×10^9^/L) ^a^	98 (65–138)	98 (65–124.5)	100 (70–149.75)	0.39
Hb (g/L) ^b^	97.05 ± 25	99.1 ± 24.67	91.55 ± 25.44	0.1
Total bilirubin (µmol/L) ^a^	61.6 (28–148.7)	49.1 (25.1–101.8)	154.15 (73.27–391.1)	<0.01
Creatinine (µmol/L) ^a^	78 (65–138)	74 (63.5–113.5)	133.5 (73–179.5)	0.01
Albumin(g/L) ^b^	27.5 ± 5.71	28.57 ± 5.76	24.5 ± 4.43	<0.01
CRP (mg/L) ^a^	20 (7.1–78.4)	10.7 (5.15–64.15)	10.7 (5.15–64.15)	<0.01
Procalcitonin (ng/L) ^a^	0.44 (0.17–0.8)	0.24 (0.1–0.7)	0.635 (0.49–0.88)	<0.01
Fibrinogen(g/L) ^a^	2.8 (1.9–3.3)	2.5 (1.85–3.2)	3.15 (2.62–3.87)	0.01
PT (s) ^a^	16.6 (14.9–20.6)	16.1 (14.75–19.6)	19.95 (16.05–26.05)	<0.01
INR ^a^	1.5 (1.3–1.9)	1.44 (1.28–1.81)	1.73 (1.45–2.4)	<0.01

^a^—median (IQR); ^b^—mean ± SD; WBC—white blood cell; Plt—platelet; Hb—hemoglobin; CRP—C-reactive protein; PT—protrombin time; INR—international normalized ratio.

**Table 3:** Infection in ALD cirrhosis.

	Total	AD	ACLF	*p*
Community-acquired infection, *n* (%)	45 (31.03)	32 (29.9)	13 (34.2)	0.07
Infection focus, *n* (%):				
UTI	21 (46.7)	18 (56.2)	3 (23.1)	
Pneumonia	14 (31.1)	8 (25)	6 (46.2)	
UTI and pneumonia	4 (8.9)	2 (6.2)	2 (15.4)	
Bacteremia	4 (8.9)	3 (9.4)	1 (7.7)	
Acute cholecystitis	1 (2.2)	1 (3.1)	/	
SBP	1 (2.2)	/	1 (7.7)	
Healthcare-associated infection, *n* (%)	63 (43.4)	41 (38.3)	22 (57.9)	0.05
Infection focus, *n* (%)				
UTI	35 (55.6)	25 (61)	10 (45.5)	
Bacteremia	20 (31.7)	10 (24.4)	10 (45.5)	
Pneumonia	5 (7.9)	4 (9.8)	1 (4.5)	
SBP	2 (3.2)	1 (2.4)	1 (4.5)	
UTI and pneumonia	1 (1.6)	1 (2.4)	/	

UTI—urinary tract infection; SBP—spontaneous bacterial peritonitis.

**Table 4:** Main causes of death.

	All Patients (*n* = 46)	AD (*n* = 24)	ACLF (*n* = 22)
Variceal bleeding, *n* (%)	7 (15.2)	4 (16.7)	3 (13.6)
Sepsis, *n* (%)	5 (10.2)	2 (8.3)	3 (13.6)
Lung failure, *n* (%)	13 (28.3)	4 (16.7)	9 (40.9)
Heart failure, *n* (%)	17 (37)	10 (41.7)	7 (31.8)
Cerebrovascular insult, *n* (%)	1 (2.2)	1 (4.2)	/
Other liver-related deaths, *n* (%)	3 (6.5)	3 (12.5)	/

**Table 5:** Predictive factors associated with 28-day mortality.

Variables	Univariate Analysis	*p*	Multivariate Analysis	*p*
	Odds Ratio (CI 95%)		Odds Ratio (CI 95%)	
HE	4.912 (1.907–12.653)	0.01	3.63 (1.068–12.340)	0.04
CAI	2.3 (1.12–4.723)	0.023		0.409
Ascites	7.836 (1.773–34.63)	0.007	6.896 (1.136–41.861)	0.036
HRS	6.040 (2.122–17.191)	0.001		0.08
SIRS	8.775 (3.784–20.348)	<0.01	9.232 (3.329–26.605)	<0.01
ACLF	7.25 (3.089–17.018)	<0.01	3.539 (1.306–9.591)	0.013

HE—hepatic encephalopathy; CAI—community-acquired infection; HRS—hepatorenal syndrome; SIRS—systemic inflammatory response syndrome; ACLF—acute-on-chronic liver failure.

**Table 6:** Baseline average values of examined scores.

Variable	All Patients	AD	ACLF	*p*
MELD ^1^	20.08 ± 8.242	17.73 ± 7.014	26.66 ± 7.913	<0.01
MELD Na ^1^	22.17 ± 8.452	19.89 ± 7.58	28.55 ± 7.5	<0.01
NLR ^1^	58 ± 5.81	5.39 ± 7.62	6.95 ± 6.409	0.265
ALBI ^1^	−1.24 ± 0.729	−1.37 ± 0.64	−0.87 ± 0.704	<0.01
LIV-IN	6.19 ± 2.93	5.63 ± 2.87	7.78 ± 2.53	<0.01
CLIF-C AD ^1^	N/A	56.23 ± 9.79	N/A	
CLIF-C ACLF ^1^	N/A	N/A	53.26 ± 7.68	

^1^—mean ± SD. AD—acute decompensation; ACLF—acute-on-chronic liver failure; MELD—model for end-stage liver disease; MELD Na—model for end-stage liver disease-sodium; NLR—neutrophil-to-lymphocyte ratio; ALBI—albumin-to-bilirubin ratio; CLIF-C AD—chronic liver failure consortium-C acute decompensation; CLIF-C-ACLF—chronic liver failure consortium-C-acute-on-chronic liver failure; LIV-IN—liver mortality inpatients; N/A-Not Assessed

**Table 7:** Clinical accuracy of LIV-IN, MELD, MELD-Na, ALBI, and NLR scores in predicting 28-day mortality.

Score	AUC	*p*	95% CI	(Cut Off)	Sensitivity (%)	Specificity (%)
LIV-IN	0.734	<0.01	0.650–0.818	5.5	75.6	53.1
MELD	0.661	0.02	0.564–0.757	16.5	75.6	44.8
MELD-Na	0.696	<0.01	0.599–0.792	21.5	71.1	58.3
ALBI	0.696	<0.01	0.604–0.788	−1.5	84.4	45.8
NLR	0.608	0.036	0.510–0.706	2.5	84.4	34.4

MELD—model for end-stage liver disease; MELD Na—model for end-stage liver disease-sodium; NLR—neutrophil-to-lymphocyte ratio; ALBI—albumin-to-bilirubin ratio; LIV-IN—liver mortality inpatients.

## Data Availability

The data presented in this study are available on request from the corresponding author. The data are not publicly available due to the privacy of the participants.
